# Occult Papillary Thyroid Carcinoma With No Identifiable Intrathyroidal Primary: A Case Report and Pooled Analysis of 23 Published Cases

**DOI:** 10.7759/cureus.112518

**Published:** 2026-07-12

**Authors:** Michael J Evans, Amanda M Sonnenburg, Elijah Elliott, Alex L Otto, Kent McIntire

**Affiliations:** 1 College of Osteopathic Medicine, Kansas City University, Joplin, USA; 2 Otolaryngology-Head and Neck Surgery, Freeman Health System, Joplin, USA

**Keywords:** braf mutation, cervical lymphadenopathy, cystic neck mass, occult primary, papillary thyroid carcinoma, radioactive iodine, shared decision-making

## Abstract

Papillary thyroid carcinoma (PTC) rarely presents as cervical lymph node metastasis without an identifiable intrathyroidal primary despite exhaustive imaging and histopathologic evaluation, representing the rarest form of occult thyroid carcinoma and posing distinct diagnostic and therapeutic challenges. We report a 27-year-old man who presented with a cystic lateral neck mass. Fine-needle aspiration confirmed metastatic PTC, yet ultrasonography, computed tomography, and positron emission tomography/computed tomography demonstrated no thyroid abnormality. Total thyroidectomy with modified radical neck dissection revealed metastatic PTC in two of the 21 lymph nodes (pN1b) without extranodal extension, while exhaustive sectioning of the thyroid demonstrated only chronic inflammatory changes without carcinoma. At three months postoperatively, thyroglobulin was undetectable with no structural evidence of disease. To contextualize this presentation, we reviewed the literature and identified 22 comparable published cases of metastatic cervical nodal PTC without an intrathyroidal primary after total thyroidectomy, yielding a pooled cohort of 23 patients, including the index case. The pooled cases showed an atypical near-equal sex distribution (52% male), frequent lateral neck presentation (65%), cystic nodal morphology in 26%, benign or normal thyroid tissue in all patients, and BRAF V600E mutations in 63% of the minority tested. At a median follow-up of 24 months, 87% had no evidence of disease. These observations are most consistent with an occult thyroid carcinoma arising from an ultramicroscopic primary tumor or a regressed primary lesion. Persistent lateral neck masses, particularly when cystic, should prompt evaluation for metastatic PTC. Total thyroidectomy with appropriate nodal dissection enables definitive diagnosis and surveillance; radioactive iodine may be deferred in selected patients with an excellent response; and overall outcomes are favorable, supporting individualized management and lifelong follow-up.

## Introduction

Papillary thyroid carcinoma (PTC) is the most common differentiated thyroid malignancy, accounting for approximately 80-85% of thyroid cancers and generally conferring an excellent prognosis [1]. A small subset of patients, however, present with occult disease in which metastatic PTC is identified despite the absence of a clinically or radiographically apparent intrathyroidal primary. Historically, the term occult papillary carcinoma referred to node-positive PTC in which the primary tumor was presumed too small to be detected by palpation or early imaging. With the advent of high-resolution ultrasonography and fine-needle aspiration biopsy (FNAB), the term has evolved to describe cases in which cervical lymph node metastasis is clinically evident, but no intrathyroidal primary is identified on imaging [2]. Although most such cases ultimately reveal a subcentimeter primary on exhaustive histopathologic sectioning, a far rarer subgroup demonstrates complete absence of thyroid carcinoma despite total thyroidectomy.

To better characterize this spectrum, Bouček et al. proposed a four-category framework encompassing presentations ranging from incidental intrathyroidal microcarcinomas to carcinoma arising within ectopic thyroid tissue [3]. Liu et al. subsequently expanded this system by introducing a fifth category describing patients with metastatic disease of unequivocal thyroid origin but no demonstrable intrathyroidal carcinoma on imaging or exhaustive pathologic evaluation [4]. This fifth category represents the rarest and most diagnostically challenging form of occult thyroid carcinoma, raising important questions regarding tumor origin, classification, and optimal management. This entity is exceptionally rare, with fewer than two dozen cases documented in the English-language literature to date. Affected patients most often present with a painless, frequently cystic lateral neck mass, a presentation that overlaps with benign cysts and other malignant causes of a cystic cervical node, including human papillomavirus (HPV)-associated oropharyngeal squamous cell carcinoma.

Here, we report the case of a 27-year-old man presenting with a cystic lateral neck mass harboring metastatic PTC, in whom no intrathyroidal primary was identified despite total thyroidectomy and exhaustive histologic evaluation. To contextualize this unusual presentation, we additionally synthesize a narrative review of 22 comparable published cases, which we integrate within the Discussion to highlight shared clinical features, diagnostic challenges, proposed pathophysiologic mechanisms, and management considerations.

## Case presentation

Patient history and initial presentation

A 27-year-old man with a history of supraventricular tachycardia (SVT) presented to his primary care physician for the evaluation of elevated blood pressure. He had discontinued metoprolol six months earlier after running out of medication and reported intermittent headaches without chest pain, palpitations, or other systemic symptoms. Metoprolol was restarted for blood pressure and rate control. At that time, there was no concern for a neck mass.

At follow-up two months later, his blood pressure remained above goal despite therapy, and physical examination demonstrated no thyroid enlargement or palpable cervical lymphadenopathy. The patient reported no history of childhood or therapeutic head-and-neck ionizing radiation exposure, no family history of thyroid carcinoma or other thyroid disease, and no known autoimmune or hormonal disorder. Hydrochlorothiazide was added, and the patient began home blood pressure monitoring.

Three months later, he returned with a newly palpable, painless mass in the left lower anterior neck, first noticed following a recent sinus infection and stable in size. Physical examination revealed a firm, approximately 2 cm lesion in the left lower neck (level IV).

Diagnostic evaluation

**Figure 1:**
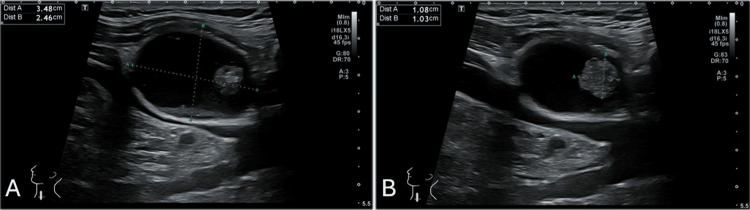
Preoperative ultrasound of the left level IV cervical region demonstrating a complex cystic lymph node. (A) Predominantly cystic component measuring approximately 3.5×3×2.5 cm. (B) Internal solid, vascularized component measuring approximately 1.1×1×1 cm, targeted on repeat fine-needle aspiration and diagnostic of papillary thyroid carcinoma

Thyroid ultrasound demonstrated a complex cystic mass in the left level IV region measuring 3.5×3×2.5 cm, containing an internal solid component measuring 1.1 cm with vascular flow (Figure 1). The thyroid gland appeared normal in size and echotexture without discrete nodules. Contrast-enhanced computed tomography (CT) of the neck confirmed a 3.7×3.3×3.2 cm complex cystic lesion without additional lymphadenopathy or thyroid nodules.

Ultrasound-guided fine-needle aspiration (FNA) was performed. Initial aspiration of the cystic component yielded non-diagnostic fluid. Repeat aspiration targeting the solid component revealed malignant cells with characteristic nuclear features of PTC in a cystic background. Thyroglobulin washout and p16/HPV immunohistochemistry were not performed at the time of FNA; although characteristic PTC cytology and subsequent thyroglobulin- and TTF-1-positive nodal staining ultimately established thyroid origin, both adjuncts could have accelerated discrimination from HPV-associated squamous cell carcinoma and represent a limitation of the initial diagnostic workup. Positron emission tomography/computed tomography (PET/CT) demonstrated no fluorodeoxyglucose (FDG)-avid lesions within the thyroid gland or elsewhere in the head and neck, further supporting the absence of an identifiable intrathyroidal primary.

Operative management

Following discussion with the patient, he underwent total thyroidectomy with left modified radical neck dissection (levels II-IV). Central neck dissection (level VI) was not performed, given the absence of clinically or radiographically evident central compartment disease and the lateral-only distribution of nodal metastases; this represents a therapeutic, compartment-oriented approach consistent with current guidelines, and the underlying rationale and supporting evidence are discussed below (see Management implications).

Preoperative laboratory studies showed mildly decreased ionized calcium (1.10 mmol/L; reference range: 1.12-1.32 mmol/L) with a normal parathyroid hormone (PTH) level. Preoperative thyroglobulin and thyroid-stimulating hormone (TSH) levels were within normal limits. Postoperatively, ionized calcium declined to 0.96 mmol/L, with preserved PTH (90.2 pg/mL), consistent with transient hypocalcemia, which was managed with oral calcium carbonate and vitamin D supplementation. He was discharged on levothyroxine 112 µg daily for TSH suppression, pending final pathology results.

Pathologic findings

Final surgical pathology revealed metastatic PTC with cystic change involving one of the two left level IV lymph nodes (1.8 cm focus) without extranodal extension. Microscopic metastasis (<1 mm) was identified in one of the 19 lymph nodes from levels II to III, also without extranodal extension. The total nodal yield was 21 nodes, with two positive nodes (pN1b). The metastatic deposits demonstrated classic papillary architecture with fibrovascular cores, nuclear grooves, pseudoinclusions, and ground-glass chromatin.

Exhaustive sectioning of the bilateral thyroid lobes (submitted entirely for microscopic examination with representative sampling at 3 mm intervals) demonstrated only mild hyperplastic changes and chronic inflammation, with no evidence of carcinoma, microcarcinoma, or papillary thyroid hyperplasia suspicious for neoplasia. No fibrosis or ectopic thyroid tissue was identified outside the thyroid capsule.

Immunohistochemical staining of the metastatic lymph nodes confirmed thyroglobulin and TTF-1 positivity, establishing unequivocal thyroid origin despite the absence of an intrathyroidal primary tumor. Molecular testing (BRAF V600E, rat sarcoma (RAS) mutations, rearranged during transfection (RET) fusions, and telomerase reverse transcriptase (TERT) promoter mutations) was not performed on the metastatic tissue, representing a missed opportunity for biologic characterization and prognostic refinement.

Postoperative course

Recovery was uncomplicated. Transient hypocalcemic symptoms (perioral paresthesias, mild hand cramping) resolved with short-term calcium and vitamin D supplementation, which was discontinued at six weeks postoperatively. Thyroid hormone replacement was titrated to achieve TSH suppression without difficulty.

At three months postoperatively, the patient reported no paresthesias, neck discomfort, voice changes, or new masses. Physical examination demonstrated well-healed surgical sites without hypertrophic scarring and no palpable lymphadenopathy. Laboratory studies showed an appropriate biochemical response to thyroidectomy, with undetectable serum thyroglobulin (<0.1 ng/mL) on levothyroxine suppression therapy and negative thyroglobulin antibodies, indicating an excellent early biochemical response at three months. Because dynamic risk stratification in differentiated thyroid carcinoma requires longer follow-up, this early response should not be interpreted as a definitive determination of low recurrence risk. Postoperative neck ultrasonography demonstrated no suspicious cervical lymphadenopathy.

## Discussion

The complete absence of an intrathyroidal primary after thyroidectomy is uncommon even within the occult PTC spectrum. To determine how the present patient compares with previously reported node-only cases, we conducted a focused review of the literature and assembled a pooled cohort that places this case in context.

Synthesis of published cases in context

This case report is accompanied by a narrative, non-systematic review with a pooled descriptive analysis; formal systematic review methodology was not applied, and the pooled observations are descriptive and hypothesis-generating rather than confirmatory. PubMed/MEDLINE was searched from database inception through January 2026 without language restriction, using combinations of ("papillary thyroid carcinoma" OR "papillary thyroid cancer") AND ("occult" OR "unknown primary" OR "no primary" OR "without primary tumor") AND ("lymph node metastasis" OR "cervical lymphadenopathy" OR "cystic neck mass"). Titles and abstracts were screened for relevance, full texts of potentially eligible reports were assessed against the criteria below, and reference lists of included articles and relevant reviews were hand-searched to identify additional cases. Cases were included if they described cervical lymph node metastasis of PTC in the absence of an identifiable intrathyroidal primary following total thyroidectomy and complete pathologic evaluation and were excluded for an identified primary, non-PTC histology, incomplete thyroidectomy, or insufficient data. Twenty-two published cases met these criteria and, with the index patient, yielded a pooled cohort of 23 cases. Continuous variables are summarized as medians with ranges and categorical variables as counts and percentages; given the small sample and reporting heterogeneity, no inferential testing was performed.

Occult thyroid carcinoma encompasses a heterogeneous spectrum of presentations, which have been variably defined in the literature [3,4]. The present pooled analysis is limited to the fifth category defined by Liu et al., comprising metastatic PTC of unequivocal thyroid origin in the absence of an identifiable intrathyroidal primary despite comprehensive imaging and histopathologic evaluation [4]. All 22 published cases and the index patient met the criteria for this category, with thyroid pathology either entirely normal or limited to non-neoplastic changes.

Across the pooled cohort, the clinical and demographic profile of Liu et al.'s Category 5 disease diverged from that of conventional PTC in several respects, as detailed for individual cases in Table 1 [2,4-15]. Patient age ranged from 17 to 76 years (median: 53), with a nearly even sex distribution (52% male and 48% female). Presentation was most often a symptomatic lateral neck mass (65%), with the remainder detected incidentally (17%) or during evaluation for thyroid-related complaints (17%); cystic nodal morphology occurred in six cases (26%) and clustered in younger patients. Nodal disease was predominantly lateral (lateral-only in 52%; any lateral involvement in 69%), with central-only in 26% and combined disease in 17%. Nodal burden was generally modest (median: 2 positive nodes; range: 1-12), the largest metastatic focus ranged widely (0.05-6.8 cm; median: 2.2 cm), and extranodal extension was documented in seven cases (30%).

**Table 1:** Clinical characteristics of the 23 cases of occult PTC ATC: anaplastic thyroid carcinoma; CT: computed tomography; ENE: extranodal extension; LN: lymph node; MVA: motor vehicle accident; ND: neck dissection; NS: not stated; PDTC: poorly differentiated thyroid carcinoma; PTC: papillary thyroid carcinoma; SCC: squamous cell carcinoma; US: ultrasound Site of metastatic foci refers to the anatomic compartment(s) involved at presentation. Positive nodes is presented as positive/total nodes examined when both were reported; NS indicates total not stated. Largest node (cm) refers to the greatest dimension of the largest metastatic focus. Individual case references are listed in the reference list; cases from multi-case source publications are numbered sequentially within each source.

Case	Study (year)	Age (y)	Sex	Presentation	Site of metastatic foci	Positive nodes	Largest node (cm)	Cystic	ENE	Thyroid pathology	Nodal histology
1	Evans et al. (2026): index case	27	M	Lateral neck mass	Lateral (left III-IV)	2/21	1.8	Yes	No	Benign thyroid disease	Classic PTC
2	Ito et al. (2008): Case 9 [2]	75	F	Lateral neck mass	Lateral	NS	4	No	Yes	Normal	Classic PTC
3	Ito et al. (2008): Case 10 [2]	64	M	Lateral neck mass	Lateral	1/NS	3.8	No	No	Normal	Classic PTC
4	Ito et al. (2008): Case 13 [2]	69	M	Incidental (CT chest, mediastinal LN)	Mediastinum	1/NS	5.5	No	No	Benign thyroid disease	Classic PTC
5	Ito et al. (2008): Case 17 [2]	17	F	Lateral neck mass	Lateral	1/NS	6.8	No	Yes	Benign thyroid disease	Classic PTC
6	Liu et al. (2014) [4]	47	M	Thyroid nodule	Central	1/NS	NS	No	No	Benign thyroid disease	Classic PTC
7	Xu et al. (2017): Case 1 [5]	49	F	Lateral neck mass	Lateral	5/11	1.9	No	No	Benign + fibrosis	Classic PTC
8	Xu et al. (2017): Case 2 [5]	53	M	Thyroid nodule	Central	1/5	1.7	No	No	Benign + fibrosis	Classic PTC
9	Xu et al. (2017): Case 3 [5]	55	M	Incidental (ND for laryngeal SCC)	Lateral + central	2/65	0.66	No	No	Benign + fibrosis	Follicular variant PTC
10	Xu et al. (2017): Case 4 [5]	74	F	Thyroid nodule (US for hyperthyroidism)	Central	2/36	0.05	No	No	Benign + fibrosis	Follicular variant PTC
11	Xu et al. (2017): Case 5 [5]	30	F	Thyroid nodule (Graves disease)	Central	1/3	0.1	No	No	Benign thyroid disease	Oncocytic solid variant PTC
12	Xu et al. (2017): Case 6 [5]	76	F	Lateral neck mass	Lateral	1/13	4.5	No	Yes	Benign + fibrosis	PDTC, columnar variant PTC
13	Xu et al. (2017): Case 7 [5]	68	M	Lateral neck mass	Lateral	6/86	5.5	No	Yes	Normal	ATC, tall-cell variant PTC
14	Agosto-Vargas et al. (2017) [6]	33	M	Incidental (CT post-MVA)	Lateral (right II-III)	3/20	1.5	Yes	No	Normal	Classic PTC
15	Carrillo et al. (2025) [7]	45	M	Lateral neck mass	Central	2/6	NS	No	No	Benign thyroid disease	Classic PTC
16	Dabas et al. (2024) [8]	38	M	Lateral neck mass	Central + lateral (left II-V)	12/75	3.0	Yes	Yes	Benign thyroid disease	Classic PTC
17	Jung et al. (2025) [9]	63	M	Lateral neck mass	Lateral (left II)	3/28	2.2	Yes	Yes	Benign thyroid disease	Classic PTC
18	Yamashita et al. (2020) [10]	66	F	Lateral neck mass	Lateral (right II)	2/23	3.8	No	Yes	Benign + fibrosis	Classic PTC
19	Chala et al. (2020) [11]	41	F	Lateral neck mass	Lateral (right II, initial); central (recurrence)	6/80	2.5	Yes	No	Normal	Classic PTC
20	Han et al. (2019) [12]	74	M	Incidental (US for vascular evaluation)	Lateral (right III)	7/21	1.8	No	No	Benign thyroid disease	Classic PTC
21	Singh et al. (2013) [13]	31	F	Lateral neck mass	Lateral (left III)	NS	NS	No	No	Normal	Classic PTC
22	Monchik et al. (2001): Case 8 [14]	29	F	Lateral neck mass	Central	1/NS	1.5	Yes	No	Fibrosis (no malignancy)	Classic PTC
23	Do et al. (2024) [15]	67	F	Lateral neck mass	Central + lateral (right IV)	2/39	NS	No	No	Benign thyroid disease	Classic PTC

The defining pathologic feature was uniformly negative thyroid tissue: evaluation after total thyroidectomy revealed benign or normal parenchyma in every case, with six cases (26%) showing entirely normal thyroid and 17 (74%) showing benign disease such as nodular hyperplasia, chronic lymphocytic thyroiditis, or adenomatous goiter, while fibrosis was documented in seven cases (30%). The diagnostic pathway showed recurring patterns: FNA was the most common initial modality but was prone to non-diagnostic sampling in cystic lesions, prompting repeat FNA, core-needle biopsy, or excisional biopsy. Thyroglobulin washout, though reported in only three cases (13%), was positive in all three. High-resolution ultrasonography identified no suspicious intrathyroidal lesion, cross-sectional imaging delineated nodal extent, PET/CT showed no metabolically active intrathyroidal disease, and immunohistochemistry, when reported, supported thyroid origin (thyroglobulin and/or TTF-1 positivity). Molecular characterization remained sparse: profiling was reported in only eight cases (35%), with BRAF V600E identified in five of those tested (63%), and broader profiling for RAS, RET, TERT promoter, and tumor protein p53 (TP53) was rarely undertaken.

Management and outcomes were similarly consistent. Total thyroidectomy was performed in all 23 cases; among the 16 reporting neck dissection details, lateral dissection was performed in 81% and central dissection in 75%, and radioactive iodine (RAI) was administered in 61%. These treatment patterns were abstracted as reported in the source publications; the rationale for individual management decisions was frequently unstated and likely reflects heterogeneity in presentation, institutional practice, and the evolution of guidelines across the 2001-2026 publication span. The controversy surrounding central neck dissection in this setting is examined further under the Management implications section.

Outcomes were favorable overall: at the last follow-up (median: 24 months; range: 3-204), 20 of the 23 patients (87%) had no evidence of disease, and three (13%) experienced recurrence. Among the remaining reported outcomes, one patient was living with persistent disease, one had died of an unknown cause, and one was lost to follow-up. These categories were not mutually exclusive: two of the three patients with recurrence (Cases 5 and 19) achieved sustained disease-free status after salvage therapy and are included in the 87% figure, whereas the third (Case 13) is still living with distant metastatic disease. Among cases reporting follow-up duration, 32% exceeded five years, and a single late recurrence at 83 months underscores the need for prolonged surveillance, as detailed in Table 2.

**Table 2:** Management and outcomes of the 23 cases of occult papillary thyroid carcinoma Bx: biopsy; CK: cytokeratin; CND: central neck dissection; FNA: fine-needle aspiration; HBME-1: Hector Battifora mesothelial-1; IHC: immunohistochemistry; ND: neck dissection; NED: no evidence of disease; NP: not performed; NS: not stated; PAX8: paired box 8; RAI: radioactive iodine; Tg: thyroglobulin; TT: total thyroidectomy; TTF-1: thyroid transcription factor 1 IHC and molecular testing were inconsistently reported across the included cases. Recurrence and outcome reflect the most recent follow-up reported in the primary source.

Case	Study (year)	Diagnostic method	IHC	BRAF V600E	Treatment: thyroid	Treatment: neck	RAI	Recurrence	Outcome	Follow-up (mo)
1	Evans et al. (2026): index case	FNA	Tg(+), TTF-1(+), Napsin A(+)	NP	TT	Lateral ND	No	No	NED	3
2	Ito et al. (2008): Case 9 [2]	FNA w/ Tg washout	NS	NP	TT	CND + lateral ND	No	No	NED	158
3	Ito et al. (2008): Case 10 [2]	FNA w/ Tg washout	NS	NP	TT	CND + lateral ND	No	No	NED	150
4	Ito et al. (2008): Case 13 [2]	Surgical path	NS	NP	TT	CND	No	No	NED	64
5	Ito et al. (2008): Case 17 [2]	FNA w/ Tg washout	NS	NP	TT	CND + lateral ND	No	Yes (at 83 mo)	NED (74 mo post-recurrence)	157
6	Liu et al. (2014) [4]	Surgical path	NS	NP	TT	CND	Yes	No	NED	NS
7	Xu et al. (2017): Case 1 [5]	FNA	NS	Positive	TT	NS	Yes	No	NED	204
8	Xu et al. (2017): Case 2 [5]	Surgical path	NS	Positive	TT	NS	No	No	NED	28.8
9	Xu et al. (2017): Case 3 [5]	Surgical path	NS	Negative	TT	NS	No	No	NED	26.4
10	Xu et al. (2017): Case 4 [5]	Surgical path	NS	Negative	TT	NS	Yes	No	NED	8
11	Xu et al. (2017): Case 5 [5]	Surgical path	NS	Negative	TT	NS	No	No	NED	8
12	Xu et al. (2017): Case 6 [5]	FNA	NS	Positive	TT	NS	Yes	No	Died (unknown cause)	44.4
13	Xu et al. (2017): Case 7 [5]	FNA	NS	Positive	TT	NS	No	Yes (distant mets: mediastinum, lung, spine)	Living with disease	24
14	Agosto-Vargas et al. (2017) [6]	Excisional Bx + FNA	NS	NP	TT	Lateral ND	Yes	NS	Lost to follow-up	NS
15	Carrillo et al. (2025) [7]	Surgical path	CK19(+), HBME-1(+)	Positive	TT	CND	Yes	No	NED	12
16	Dabas et al. (2024) [8]	FNA	NS	NP	TT	CND + lateral ND	Yes	No	NED	12
17	Jung et al. (2025) [9]	Open Bx	PAX8(+)	NP	TT	CND + lateral ND	Yes	No	NED	3
18	Yamashita et al. (2020) [10]	Core-needle Bx	TTF-1(+), Tg(+)	NP	TT	CND + lateral ND	Yes	No	NED	24
19	Chala et al. (2020) [11]	FNA	Tg(+), TTF-1(+), CK7(+), CK20(–)	NP	TT	Lateral ND	Yes	Yes (central neck, 1 y)	NED	NS
20	Han et al. (2019) [12]	FNA	NS	NP	TT	Lateral ND	Yes	No	NED	6
21	Singh et al. (2013) [13]	Excisional Bx	Tg(+)	NP	TT	CND + lateral ND	Yes	No	NED	3
22	Monchik et al. (2001): Case 8 [14]	Open Bx	Tg(+)	NP	TT	CND + lateral ND	Yes	No	NED	96
23	Do et al. (2024) [15]	FNA	NS	NP	TT	CND + lateral ND	Yes	No	NED	NS

Comparison of the index case with the pooled cohort

A structured comparison of the index case with the pooled cohort is summarized in Table 3. The patient was younger than the cohort median (27 vs. 53 years) and presented with a cystic lateral neck mass. Nodal disease was confined to the lateral compartment with low nodal burden and no extranodal extension, aligning with favorable-risk features observed in the majority of cases. Thyroid pathology demonstrated benign inflammatory changes without fibrosis, consistent with reported benign findings but without features suggestive of regression. Early postoperative findings demonstrated no evidence of disease, consistent with the favorable outcomes observed across the cohort, though follow-up remains limited.

**Table 3:** Comparison of the index case with the pooled cohort of occult PTC (N=23) CLND: central lymph node dissection; ENE: extranodal extension; FNA: fine-needle aspiration; IHC: immunohistochemistry; ND: neck dissection; NED: no evidence of disease; PTC: papillary thyroid carcinoma; RAI: radioactive iodine; Tg: thyroglobulin; TTF-1: thyroid transcription factor 1; US: ultrasound All findings from the pooled cohort are descriptive and hypothesis-generating. Denominators vary by variable because not all published cases reported every data element; where this occurs, the reporting subset (n) is indicated. The Alignment column summarizes whether the index case falls within, above, or below the cohort distribution; it does not imply statistical testing.

Characteristic	Index case (Evans et al. (2026))	Pooled cohort (N=23)	Alignment of index case vs. cohort
Demographics			
Age at diagnosis	27 years	17-76 y; mean: 51.8±18.3; median: 53	Younger than the cohort median; in young adult tier (22%)
Sex	Male	52% male (12/23); 48% female (11/23)	Consistent with the cohort's atypical near-equal ratio
Clinical presentation			
Presenting feature	Symptomatic cystic lateral neck mass (left level IV)	Lateral neck mass: 65% (15/23); thyroid-related: 17% (4/23); incidental: 17% (4/23)	Consistent with the modal (most common) presentation
Cystic nodal morphology	Yes	26% (6/23); more frequent in younger patients (mean age: 38.5 y)	Aligns with the younger cystic subgroup
Nodal disease			
Nodal compartment distribution	Lateral only (left levels II-IV)	Lateral-only: 52% (12/23); central-only: 26% (6/23); lateral + central: 17% (4/23); mediastinal: 4% (1/23); any lateral involvement: 69% (16/23)	Matches the most common (lateral-only) pattern
Number of positive nodes	2 of 21 (pN1b)	Median: 2; range: 1-12 (n=21 reporting)	At the cohort median
Size of the largest metastatic focus	1.8 cm	Median: 2.2 cm; range: 0.05-6.8 (n=19 reporting)	Below the cohort median
ENE	Absent	30% (7/23)	Favorable; in the ENE-negative majority
Thyroid pathology (post-thyroidectomy)			
Thyroid parenchymal findings	Mild hyperplasia with chronic inflammation; no carcinoma, microcarcinoma, or fibrosis identified	Normal: 26% (6/23); benign thyroid disease: 74% (17/23); fibrosis present in 30% (7/23)	Consistent with the benign thyroid disease subgroup; no fibrosis
Nodal histologic variant	Classic PTC	Classic PTC predominant; follicular, oncocytic, columnar, and tall-cell variants reported in the minority	Consistent with the modal nodal histology
Molecular profile			
BRAF V600E status	Not performed	Tested in 35% (8/23); positive in 63% of tested (5/8)	Knowledge gap; not assessed in index case
Other molecular testing (RAS, RET, TERT, TP53)	Not performed	Rarely reported across the cohort	Knowledge gap; consistent with cohort-wide underreporting
Surgical management			
Thyroid surgery	Total thyroidectomy	Total thyroidectomy in 100% (23/23)	Consistent with universal cohort practice
Neck dissection	Left modified radical neck dissection (levels II-IV); central neck dissection not performed	Among 16 cases reporting details: lateral ND: 81% (13/16); central ND: 75% (12/16)	Departure from cohort norm: CLND omitted
Adjuvant RAI	Deferred (response-adapted surveillance)	Administered in 61% (14/23); deferred in 39% (9/23)	Minority approach; consistent with response-adapted care
Outcome			
Postoperative thyroglobulin	Undetectable (<0.1 ng/mL) on suppression; negative Tg antibodies	Not systematically reported; undetectable Tg associated with favorable outcomes in reporting series	Indicates excellent response to therapy
Recurrence	None	13% (3/23); single late recurrence at 83 months	No recurrence at early follow-up
Status at the last follow-up	NED	NED: 87% (20/23); recurrence: 13% (3/23); living with the disease: 4% (1/23); died of unknown cause: 4% (1/23); lost to follow-up: 4% (1/23)	Consistent with the favorable outcome majority
Duration of follow-up	3 months	Median: 24 months; range: 2-204 (n=19 reporting)	Short follow-up; lifelong surveillance warranted

Classification and cohort interpretation

These cases conform to Liu et al.'s fifth category of occult thyroid carcinoma-metastatic PTC of unequivocal thyroid origin without a demonstrable intrathyroidal primary despite comprehensive imaging and exhaustive histopathologic evaluation [4]. Four features recur across the pooled cohort and frame the interpretation that follows: (1) heterogeneous clinical presentation across both sexes, a broad age range, and varied modes of detection; (2) uniformly negative thyroid pathology after total thyroidectomy; (3) limited molecular characterization, dominated by BRAF V600E assessment; and (4) generally favorable outcomes tempered by rare late recurrences. Liu et al.'s Category 5 does not appear to constitute a distinct molecular entity but provides a useful clinical framework for a presentation that falls outside conventional PTC staging and risk models.

The near-equal sex distribution in the cohort contrasts with the approximately 3:1 female predominance reported in conventional PTC. This atypical ratio is hypothesis-generating and may reflect differences in tumor biology, immune-mediated regression, or referral patterns; definitive conclusions cannot be drawn from a descriptive analysis of this size. The documentation of autoimmune thyroid disease in a subset of cases raises the possibility of an association between immune activation and primary tumor regression, a hypothesis previously advanced in thyroid cancer literature. Cystic nodal morphology was most often observed in younger patients (Table 4). This pattern is diagnostically relevant, as such lesions may mimic benign branchial cleft cysts and delay definitive diagnosis.

**Table 4:** Cohort summary statistics (N=23) ATC: anaplastic thyroid carcinoma; BRAF: B-Raf proto-oncogene; cN1a: central nodal metastasis; cN1b: lateral nodal metastasis; ENE: extranodal extension; NED: no evidence of disease; PDTC: poorly differentiated thyroid carcinoma; PTC: papillary thyroid carcinoma; RAI: radioactive iodine; RAS: rat sarcoma viral oncogene homolog; RET: rearranged during transfection proto-oncogene; SD: standard deviation; TERT: telomerase reverse transcriptase; TP53: tumor protein p53 All values are descriptive and hypothesis-generating; no inferential statistics were performed. For variables incompletely reported across the included publications, denominators reflect the number of cases with data available (n) rather than the full cohort of 23. Mean±SD is retained alongside median (range) for continuous variables to facilitate cross-study comparison; median (range) is the recommended summary given the non-normal distribution of nodal size and number.

Domain/parameter	Value
Demographics	
Age at diagnosis, years	Median: 53 (range: 17-76); mean: 51.8±18.3 (n=23)
Sex, male	52% (12/23)
Sex, female	48% (11/23)
Clinical presentation	
Symptomatic lateral neck mass	65% (15/23)
Identified during thyroid-related workup	17% (4/23)
Incidental finding	17% (4/23)
Cystic nodal morphology	26% (6/23); mean age of the cystic subgroup: 38.5 y
Nodal disease characteristics	
Nodal distribution: lateral-only	52% (12/23)
Nodal distribution: lateral + central	17% (4/23)
Nodal distribution: central-only	26% (6/23)
Nodal distribution: mediastinal only	4% (1/23)
Any lateral involvement (cN1b)	69% (16/23)
Size of the largest metastatic focus, cm	Median: 2.2 (range: 0.05-6.8); mean: 2.8±1.9 (n=19)
Number of positive lymph nodes	Median: 2 (range: 1-12); mean: 3.0±2.8 (n=21)
ENE	30% (7/23)
Thyroid pathology (post-thyroidectomy)	
Entirely normal thyroid parenchyma	26% (6/23)
Benign thyroid disease (any non-neoplastic finding)	74% (17/23)
Fibrosis reported	30% (7/23)
Nodal histology	
Classic PTC	Predominant histology across the cohort
Variants (follicular, oncocytic, columnar, tall-cell; 2 cases with PDTC or ATC components)	Minority of cases
Molecular profile	
BRAF V600E testing performed	35% (8/23)
BRAF V600E positive (among tested)	63% (5/8)
Other testing (RAS, RET, TERT, TP53)	Rarely reported
Surgical management	
Total thyroidectomy	100% (23/23)
Lateral neck dissection (among cases reporting details)	81% (13/16)
Central neck dissection (among cases reporting details)	75% (12/16)
Adjuvant therapy	
RAI administered	61% (14/23)
RAI deferred	39% (9/23)
Outcomes at the last follow-up	
NED	87% (20/23)
Recurrence	13% (3/23); single late recurrence at 83 months
Living with the disease	4% (1/23)
Died of unknown cause	4% (1/23)
Lost to follow-up	4% (1/23)
Follow-up duration, months	Median: 24 (range: 2-204) (n=19)

Pathophysiologic considerations

Several mechanisms may explain PTC presenting as cervical lymph node metastasis without an identifiable intrathyroidal primary. The most widely supported explanation is the presence of an ultramicroscopic primary tumor below histologic sampling thresholds. Even when the entire thyroid gland is submitted for evaluation, only approximately 0.1% is examined microscopically, as each slide represents a 4 µm section from a 3 mm block [5]. Consequently, microcarcinomas well within the size range capable of producing nodal metastases may evade detection [16,17]. This mechanism is consistent with our patient's presentation and the majority of cases in our cohort, including those with entirely normal thyroid parenchyma (26%).

A second mechanism is partial or complete spontaneous regression of an intrathyroidal primary. Although rare (estimated incidence: ~1 in 140,000 across malignancies [18]), regression-associated features such as fibrosis, venulitis, and isolated microscopic tumor remnants have been reported in metastatic PTC without an identifiable primary [19,20], with proposed mechanisms including immune-mediated clearance and cytokine-driven remodeling [21]. Macrophage-mediated phagocytosis of thyroid tumor cells has been directly observed and is paradoxically associated with aggressive features, including nodal metastasis and extrathyroidal extension [22]. Fibrosis in roughly one-third of cohort cases may be consistent with an immune-mediated regression spectrum in some patients, although heterogeneous reporting precludes firm conclusions. Chronic inflammation, such as that observed in the index patient, is a nonspecific finding and does not by itself establish tumor regression; notably, the absence of fibrosis in the index case argues against a regressed primary and instead favors an ultramicroscopic focus below histologic sampling thresholds.

Malignant transformation of ectopic thyroid tissue represents a third but far less common mechanism. Ectopic thyroid tissue typically occurs along the thyroglossal duct tract, with malignant transformation reported in approximately 1% of cases. Lateral neck ectopic thyroid carcinoma is exceedingly rare and reported only sporadically [6,23]. Definitive diagnosis requires excluding intrathyroidal carcinoma and demonstrating an independent vascular supply, both of which are rarely feasible intraoperatively.

Taken together, missed microcarcinoma and partial regression appear to account for most cases, with ectopic-origin carcinoma a rare alternative; fibrosis seen in 30% of cases in the cohort may reflect a regressed or "burned-out" primary in a subset. In the present case, the absence of fibrosis favors an ultramicroscopic primary rather than a regressed lesion, though this distinction cannot be made definitively.

Diagnostic considerations

The principal diagnostic challenge in occult thyroid carcinoma lies not in confirming thyroid origin but in explaining the absence of an intrathyroidal primary and in achieving an accurate preoperative diagnosis of nodal disease, as the index case illustrates.

A lateral neck mass was the most common presenting feature in our cohort (65%), with cystic nodal morphology in 26% of cases. Cystic nodal metastases account for 6.7-13% of PTC nodal disease and frequently mimic benign branchial cleft cysts in younger patients [24], broadening the differential to include congenital cysts, inflammatory lymphadenitis, and metastases from other head-and-neck primaries, most notably HPV-associated oropharyngeal squamous cell carcinoma, which characteristically presents as a cystic lateral neck node, as well as salivary gland and nasopharyngeal carcinomas.

Sonographic features suggesting nodal metastasis include loss of the fatty hilum, rounded morphology, microcalcifications, and peripheral vascularity; cystic change is particularly suspicious when accompanied by solid components or calcifications [25]. Cross-sectional CT or magnetic resonance imaging (MRI) delineates the extent of disease, whereas PET/CT has limited sensitivity for small nodal disease.

FNA remains the preferred initial modality but is limited by non-diagnostic sampling, most often from cystic degeneration, necrosis, or sampling error. The American Thyroid Association (ATA) 2025 guidelines recommend targeted aspiration of solid components and adjunctive thyroglobulin washout in cystic or indeterminate nodes [25]; washout demonstrates high sensitivity and specificity for thyroid origin and was uniformly positive when performed in our cohort (3/3). Because a cystic lateral neck node in an adult may alternatively represent HPV-associated oropharyngeal squamous cell carcinoma, p16 immunohistochemistry or high-risk HPV testing should be obtained alongside thyroglobulin washout when cytology is not definitively diagnostic, thereby enabling early discrimination between thyroid and oropharyngeal origins. When FNA is non-diagnostic, core-needle or excisional biopsy may be required, the latter favored when accessibility and therapeutic intent support upfront resection.

Following the confirmation of metastatic PTC, a comprehensive thyroid evaluation is essential, yet ultrasound and cross-sectional imaging cannot exclude ultramicroscopic disease. Definitive exclusion of an intrathyroidal primary ultimately requires thyroidectomy with exhaustive histopathologic evaluation, though sampling limitations persist even with complete submission [5]. Immunohistochemical staining for thyroglobulin and TTF-1 remains essential to confirm thyroid origin when cytologic features are equivocal.

The median size of the largest metastatic focus exceeds that typically seen in PTC nodal metastases, possibly reflecting a delayed diagnosis without a palpable primary. Extranodal extension, traditionally a high-risk feature, was documented in a minority of cases but did not consistently predict adverse outcomes in this descriptive cohort.

Management implications

Management of metastatic PTC without an identifiable primary is informed by case-based evidence and established thyroid cancer guidelines, with necessary adaptation to account for occult presentation.

Across published reports, total thyroidectomy was performed universally, consistent with ATA recommendations for clinically apparent nodal metastases (cN1) [25]. It removes potential ultramicroscopic foci, enables reliable thyroglobulin surveillance, supports RAI therapy when indicated, and permits definitive histopathologic assessment of the gland.

Nodal management follows standard compartment-oriented principles. For lateral neck disease (cN1b), therapeutic dissection of levels II-IV (and Vb when indicated) is recommended, as limited excision increases the risk of persistent disease [25]. The index patient underwent a modified radical neck dissection consistent with these principles. The role of central lymph node dissection (CLND) remains more nuanced. Although ATA 2025 recommends prophylactic CLND in patients undergoing lateral neck dissection [25], its benefit in occult PTC is uncertain and must be balanced against the risks of hypoparathyroidism and recurrent laryngeal nerve injury [25,26]. A substantial proportion of cohort patients with lateral-only disease did not undergo CLND despite guideline recommendations, reflecting heterogeneity in presentation, evolving surgical paradigms, and ongoing controversy.

In the index case, the decision to omit CLND was made intraoperatively on the basis of the absence of clinically or radiographically evident central compartment disease and the lateral-only distribution of nodal metastases, consistent with a therapeutic rather than prophylactic compartment-oriented approach [25]. The subsequently observed excellent early postoperative biochemical response provides retrospective support for the adequacy of this approach, but did not inform the intraoperative decision. This individualized strategy is further supported by evidence that residual nodal disease more often reflects incomplete compartmental dissection than the omission of prophylactic surgery [26].

Postoperative RAI decision-making is similarly complex. ATA 2025 recommends a risk-adapted approach, but the absence of a defined primary complicates traditional stratification in occult PTC. RAI utilization in our cohort was heterogeneous, with comparable outcomes observed in series that omitted RAI entirely and those that used it routinely [2,5]. These patterns reflect the paucity of occult PTC-specific evidence and reliance on extrapolation from conventional PTC data. Factors most relevant to RAI decision-making include age, nodal burden, extranodal extension, completeness of resection, and postoperative thyroglobulin, with undetectable thyroglobulin the strongest predictor of excellent outcome. In the index patient, RAI was deferred following multidisciplinary discussion, with RAI reserved for biochemical or structural recurrence. This approach aligns with contemporary guideline emphasis on individualized, response-adapted care [25].

Molecular testing represents a further management consideration, although its role in this setting is currently limited. Molecular characterization of Liu et al.'s Category 5 disease remains a major knowledge gap. Among tested cases, BRAF V600E prevalence appeared broadly comparable to rates reported in conventional PTC [27], suggesting that molecular pathogenesis may parallel that of conventional PTC rather than represent a distinct occult subtype; this inference, however, is limited by the small number of tested cases. Other relevant alterations, including TERT promoter mutations, RAS mutations, RET fusions, and TP53 mutations, each carry distinct prognostic and therapeutic implications but are rarely reported in this setting. Broader profiling may be most informative in patients with adverse features or recurrent disease; its incremental value for upfront decision-making in patients with favorable clinical features, such as the index case, is likely limited.

Prognostic interpretation

Overall prognosis for Liu et al.'s Category 5 disease appears favorable (Table 4). The cohort's recurrence rate compares favorably with conventional N1b PTC, in which five-year recurrence rates of 20-30% have been reported [28]. Traditional high-risk features such as extranodal extension and bulky nodal disease did not uniformly predict adverse outcomes in this descriptive cohort: extranodal extension was documented in seven cases (30%), of which only two experienced recurrence. Nonetheless, the adverse outcomes that did occur clustered among cases with high-risk features, most notably Case 13, which harbored bulky mediastinal disease and remains the only patient living with distant metastatic disease, indicating that these features retain prognostic relevance even when they do not uniformly predict outcome. Given the small and heterogeneous sample, these observations are hypothesis-generating and should not be construed as diminishing the established prognostic weight of extranodal extension or nodal bulk.

Younger age likely contributes to a favorable risk interpretation, consistent with conventional PTC data and American Joint Committee on Cancer (AJCC) Eighth Edition staging [29]. Outcomes also parallel those reported for papillary thyroid microcarcinoma, the presumed source of many occult primaries, in which nodal metastases are common but long-term survival remains excellent. An excellent response to initial therapy, defined as undetectable thyroglobulin and negative imaging, remains the strongest predictor of durable remission [29,30].

The index patient's undetectable postoperative thyroglobulin and negative imaging appear consistent with the cohort's low-risk majority. Dynamic risk stratification based on response to therapy supports the consideration of surveillance without RAI in carefully selected patients with excellent initial response while acknowledging the importance of lifelong follow-up given rare late recurrences. Taken together, these observations suggest that Liu et al.'s Category 5 disease may represent a distinct clinical presentation with generally indolent behavior rather than a separate molecular entity. However, interpretation remains tentative given the limitations of descriptive, case-based data.

Limitations

This synthesis was conducted as a narrative rather than a systematic review and is subject to selection and publication bias, heterogeneous reporting, and variable diagnostic workups. Pooled frequencies are therefore descriptive and hypothesis-generating; they should not be interpreted as prevalence or effect estimates, and formal between-group comparisons (e.g., RAI versus no RAI, extranodal extension versus no extranodal extension) cannot be supported with this dataset. Because the included publications span 2001-2026, pooled estimates aggregate cases across substantial evolution in ultrasound resolution, pathologic sectioning, and molecular assays; data were not adjusted for publication era, and the true prevalence of Liu et al.'s Category 5 disease may be lower under contemporary evaluation. Definitive distinction between missed ultramicroscopic carcinoma, spontaneous regression, and rare ectopic-origin carcinoma is not currently possible within this framework. The index case is further limited by a follow-up period of only three months, which is sufficient to demonstrate an early biochemical response but not to establish durable remission. Additionally, molecular testing was not performed on the metastatic lymph node, preventing risk stratification based on BRAF V600E, TERT promoter, RAS, and RET alterations. Finally, the comprehensive diagnostic workup described here may not be universally accessible, raising health equity considerations for generalization.

## Conclusions

PTC can rarely present as metastatic cervical lymphadenopathy without an identifiable intrathyroidal primary, despite exhaustive evaluation, a pattern captured by Liu et al.'s fifth category of occult thyroid carcinoma and most plausibly explained by an ultramicroscopic primary below sampling thresholds or partial regression of an intrathyroidal primary. Persistent lateral neck masses, particularly when cystic, warrant a high index of suspicion for metastatic PTC, careful ultrasound assessment, targeted FNA of solid components, and adjunctive thyroglobulin washout testing to reduce false-negative evaluations. Once metastatic PTC is confirmed, total thyroidectomy with compartment-oriented nodal dissection provides definitive thyroid assessment, enables thyroglobulin surveillance, and preserves the option of RAI when indicated. Outcomes across published cases were favorable overall, but not uniformly so: the recurrences and the single case of persistent distant disease clustered among patients with extranodal extension and bulky nodal disease, indicating that these established high-risk features retained prognostic relevance even in this small cohort. The index patient's excellent early biochemical response is consistent with the favorable-risk majority, although the three-month follow-up precludes any conclusion about durable remission. Rare late recurrences warrant lifelong surveillance.

Because this analysis is descriptive and based on a small number of heterogeneous published cases, all findings, including any apparent association between younger age and favorable outcome, should be regarded as hypothesis-generating only and not as evidence of an effect. They nonetheless provide a framework for diagnosis, risk interpretation, and management when guideline-directed care must be adapted to an occult primary. Future progress will depend on multicenter registries with standardized reporting, systematic molecular profiling, long-term follow-up, and incorporation of patient-reported outcomes to refine biologic classification, guide radioactive iodine use, and optimize survivorship-centered care. Priority research directions include prospective registries with uniform molecular testing, comparative studies of radioactive iodine versus surveillance in intermediate-risk disease, mechanistic studies of primary regression, and health services research addressing cost-effectiveness and access to specialized thyroid cancer care.
